# Adaptive Immune Response to *Mycobacterium abscessus* Complex (MABSC) in Cystic Fibrosis and the Implications of Cross-Reactivity

**DOI:** 10.3389/fcimb.2022.858398

**Published:** 2022-04-20

**Authors:** Renan Marrichi Mauch, Peter Østrup Jensen, Tavs Qvist, Mette Kolpen, Claus Moser, Tacjana Pressler, Marcos Tadeu Nolasco da Silva, Niels Høiby, The Copenhagen Study Group on Mycobacterial Infections in Cystic Fibrosis, Daniel Faurholt-Jepsen

**Affiliations:** ^1^ Center for Investigation in Pediatrics, School of Medical Sciences, University of Campinas, Campinas, Brazil; ^2^ Clinical Microbiology Department, Rigshospitalet (Copenhagen University Hospital), Copenhagen, Denmark; ^3^ Costerton Biofilm Center, Department of Immunology and Microbiology, Faculty of Health and Medical Sciences (Panum Institute), University of Copenhagen, Copenhagen, Denmark; ^4^ Institute of Inflammation Research, Rigshospitalet (Copenhagen University Hospital), Copenhagen, Denmark; ^5^ Cystic Fibrosis Adult Clinic , Rigshospitalet (Copenhagen University Hospital), Copenhagen, Denmark

**Keywords:** Nontuberculous Mycobacteria, *Mycobacterium abscessus* Complex, cystic fibrosis, cellular immunology, cytokines, flow cytometry

## Abstract

**Background:**

We aimed to characterise the adaptive immune response to *Mycobacterium abscessus* complex (MABSC) and its cross-reactivity with *Mycobacterium avium* complex (MAC) and *Mycobacterium bovis* (Bacille Calmette-Guérin, BCG) in cystic fibrosis (CF) patients and non-CF controls in terms of lymphocyte proliferation and immunophenotyping, cytokine production and anti-MABSC IgG plasma levels.

**Methods:**

In this cross-sectional analysis, peripheral blood mononuclear cells (PBMC) from CF patients with MABSC (CF/MABSC, n=12), MAC infection history (CF/MAC, n=5), no NTM history (CF/NTM-, n=15), BCG-vaccinated (C/BCG+, n=9) and non-vaccinated controls (C/BCG-, n=8) were cultured for four days under stimulation with an in-house MABSC lysate and we used flow cytometry to assess lymphocyte proliferation (given by lymphoblast formation) and immunophenotypes. Cytokine production was assessed after overnight whole blood stimulation with the same lysate, and anti-MABSC IgG levels were measured in plasma from non-stimulated blood.

**Results:**

All CF/MABSC patients had increased CD3+ and CD19+ lymphoblast formation upon PBMC stimulation with MABSC lysate. There was a higher rate of CD3+ than CD19+ lymphoblasts, predominance of CD4+ over CD8+ lymphoblasts, IFN-γ, TNF-α and IL-2 production, low production of the Th17-associated IL-17, and discrete or no production of Th2/B cell-associated cytokines soluble CD40 ligand (CD40L), IL-4 and IL-5, indicating a Th1-dominated phenotype and infection restricted to the lungs. A similar pattern was seen in C/BCG+ controls, and CF/MAC patients, pointing to cross-reactivity. MABSC-IgG levels were higher in CF/MABSC patients than in both control groups, but not CF/NTM- patients, most of whom also had CD3+ and/or CD19+ lymphoblast formation upon PBMC stimulation, indicating previous exposure, subclinical or latent infection with MABSC or other NTM.

**Conclusion:**

The anti-MABSC immune response is Th1-skewed and underlines the cross-reactivity in the anti-mycobacterial immune response. The results, together with published clinical observations, indicate that BCG vaccination may cross-react against NTM in CF patients, and this should be investigated. Due to cross-reactivity, it would also be interesting to investigate whether a combination of MABSC-induced cytokine production by blood cells and anti-MABSC IgG measurement can be useful for identifying latent or subclinical infection both with MABSC and other NTM in CF patients.

## Introduction

Among nontuberculous mycobacteria (NTM), the *Mycobacterium abscessus* complex (MABSC) is a major concern for patients with cystic fibrosis (CF), due to its pathogenicity and association with rapid deterioration of lung function and early death (Nessar et al., 2012; Qvist et al., 2015a; Degiacomi et al., 2019). A retrospective 13-year study performed by our group at the Copenhagen CF centre found that less than a third of CF patients with MABSC infection treated with antibiotics were able to clear the infection and one-fourth eventually underwent lung transplantation or died (Qvist et al., 2015a). Furthermore, MABSC infection had the most pronounced effect in the deterioration of lung function, even when compared to well-known CF respiratory pathogens, including *Pseudomonas aeruginosa* (Qvist et al., 2016). MABSC can also form biofilm in the lungs, which explains why eradication therapy is rarely successful (Kwak et al., 2019; Pereira et al., 2020).

The immune response to mycobacterial infections in CF is multifaceted, comprising mucociliary clearance (impaired in CF (Elborn, 2016)) and a major role of CD4 T cells that produce the macrophage-activating cytokine interferon-gamma (IFN-γ), and tumour necrosis factor alpha (TNF-α) – involved in granuloma formation (Richards and Olivier, 2019; Pereira et al., 2020). We have previously shown that serum anti-MABSC IgG antibodies rise before and during clinical infection and can be used for diagnosis, although their protective capacity is unclear, which is also the case in *P. aeruginosa* infection. In some cases, MABSC IgG rose several months or years before MABSC isolation in culture (Qvist et al., 2015b), indicating latent or subclinical infection, which is well-known to be the case for *Mycobacterium tuberculosis* (Oh et al., 2020), and was also the seen in CF patients with MAC infection (Qvist et al., 2017; Ravnholt et al., 2019). We also found cross-reactivity between MABSC and other NTM when measuring either anti-MABSC or anti-MAC serum IgG (Qvist et al., 2017; Ravnholt et al., 2019), in accordance with the genetic relatedness of NTM species (Pereira et al., 2020). Interestingly, a study showed that both the presence of *Mycobacterium tuberculosis* infection and the Bacillus Calmette-Guérin (BCG) vaccine (composed of an inactivated *Mycobacterium bovis* strain), induced cross-reactive lymphocyte response against MABSC and MAC in mice and humans (Abate et al., 2019). The influence of BCG in the anti-MABSC response has not been broadly assessed in the CF clinical setting. However, we found that NTM infection is not a major issue in one of the main CF centres in Brazil, where BCG vaccination is routinely given to infants (including CF patients) in their first month of life (Aiello et al., 2018). In contrast, MABSC infection is an increasing problem at the Copenhagen CF Centre, in Denmark, where general BCG vaccination was discontinued in 1986, with NTM rarely being seen before that year (Hjelt et al., 1994). Studying the pathogen-host interaction in MABSC infection is important to understand the natural history of the infection, which, in turn, can help to drive early suitable diagnostic and therapeutic approaches for it. Also, investigating cross-reactive responses between MABSC and other mycobacteria, including *M. tuberculosis* and *M. bovis*, could clarify to which extent a response directed to a bacterial group can target another.

Our aim in the present study was to describe the main characteristics of the adaptive immunity to MABSC in CF patients with a history of MABSC infection, using assays based on flow cytometry, cytokine, and antibody measurement. Studying the T and B cell immunity can reveal whether a T-B cell polarisation is present in MABSC lung infection in CF, as we have shown to be the case in *P. aeruginosa* infection (Moser et al., 2000), and also in *M. avium* subsp. *paratuberculsosis* infection in patients with inflammatory bowel disease (Collins et al., 2000). If this is the case, testing for both IgG and cytokine release response could improve the diagnosis of subclinical and latent infection with MABSC and possibly other NTM in CF. We also investigated whether non-CF healthy controls vaccinated with BCG respond to cell stimulation with MABSC.

## Methods

### Inclusion Criteria

The CF patients who participated in the present study are followed once a month at the CF adult clinic, Copenhagen CF Centre (Rigshospitalet), and have respiratory samples collected for microscopy, bacterial (including mycobacterial) and fungi culture at every visit to the clinic. We enrolled 32 individuals with CF, with a median age of 27.5 years (range: 20–56), who were classified into three groups according to their record of respiratory microbiological culture results: Patients with history of MABSC infection (CF/MABSC, n=12), patients with history of MAC infection (CF/MAC, n=5) and patients without history of NTM infection (CF/NTM-, n=15). At least one culture positive for MABSC or MAC was considered for this classification (**Supplementary Table 1**). All individuals in the CF/MABSC and CF/MAC groups had at one point fulfilled the American Thoracic Society and Infectious Diseases Society of America (ATS/IDSA) criteria for NTM infection (Griffith et al., 2007). Based on routine microbiological respiratory culture for NTM, seven individuals had MABSC or MAC infection for up to one year and cleared it; five had MABSC or MAC infection for 2-5 years, three out of whom had cleared it and two had repeated positive MABSC or MAC cultures by the time when the study started; five had MABSC or MAC infection for more than five years, two of whom cleared it and three had repeated positive MABSC or MAC cultures by the time that the study started (**Supplementary Table 2**). Seven CF patients were vaccinated with BCG, three of whom were in the CF/MABSC group, two were in the CF/MAC group and two were in the CF/NTM- group (**Supplementary Table 1**). All this information was retrieved after all results were available, meaning that the main author, who conducted the lab analyses, was blinded to the NTM history and all other characteristics of the enrolled patients.

For comparison, we included a control group of non-CF individuals with no documented history of NTM infection, composed of students and staff from the University of Copenhagen and Rigshospitalet. The controls were further classified according to their BCG vaccination status as individuals who were vaccinated with BCG (C/BCG+) and individuals who were not vaccinated with BCG (C/BCG-) (**Table 1** and **Supplementary Table 1**). The vaccination status was self-reported by the controls after reviewing their vaccination records. The non-CF control group consisted of 17 individuals, 10 of whom were male, with median age of 32 years old (range: 23–78). Nine controls were vaccinated with BCG and eight were not (**Supplementary Table 1**). Approximately 20 mL of blood were drawn from all volunteers in heparinised tubes for the assays.

**Table 1:** Data of the groups enrolled in the study: CF/MABSC (CF patients with history of MABSC), CF/MAC (CF patients with history of MAC infection), CF/NTM- (CF patients without history of NTM infection), C/BCG+ (non-CF controls vaccinated with BCG), and C/BCG- (Non-CF controls who were not vaccinated with BCG). Rate of total lymphoblasts formed after PBMC stimulation with MABSC lysate (after subtracting background lymphoblast formation), rate of different cell subtypes within the formed lymphoblasts, concentration of interferon-gamma (IFN-γ), tumour necrosis factor-alpha (TNF-α), soluble CD40 ligand (CD40L), interleukin (IL)-2, IL-4, IL-5 and IL-17 in plasma after overnight whole blood stimulation with MABSC lysate, and levels of anti-MABSC IgG in plasma from unstimulated whole blood.

Variables/Groups	Median values (Minimum - Maximum; IQR)				
Frequency of individuals with increased lymphoblast formation upon PBMC challenge with MABSC	CF/MABSC (n = 12)	CF/MAC (n = 5)	CF/NTM-(n = 15)	C/BCG+(n = 9)	C/BCG-(n = 8)
	N/Total (%): 12/12 (100.0)	N/Total (%): 4/5 (80.0)	N/Total (%): 10/15 (66.7)	N/Total (%): 8/9 (88.9)	N/Total (%): 3/8 (37.5)
***% Lymphoblasts within T cells***	1.53 (0.0 – 29.8; 2.7)	1.42 (0.0 – 4.4; 3.4)	0.51 (0.0 – 9.5; 4.0)	2.24 (0.0 – 8.9; 3.6)	0.00 (0.0 – 2.5; 1.1)
%CD4+	40.5 (19.8–66.6; 29.3)	46.7 (36.5–57.7; 17.0)	45.5 (28.0–73.0; 11.8)	54.4 (23.1–83.5; 27.2)	NI^1^
*%CD45RO+*	83.4 (62.5–92.7; 7.0)	84.8 (61.8–91.5; 24.3)	92.3 (81.8–98.1; 3.0)	88.9 (65.3–97.6; 23.4)	NI^1^
%CD8+	11.5 (5.9–28.8; 15.8)	16.6 (8.8–57.7; 20.1)	9.2 (6.9–14.9; 2.7)	13.0 (7.5–36.2; 17,2)	NI^1^
*%CD45RO+*	54.3 (29.2–80.9; 20.3)	53.7 (29.0–75.0; 36.1)	64.2 (30.1–72.2; 11.3)	60.8 (36.0–85.9; 29.9)	NI^1^
***% Lymphoblasts within B cells***	2.25 (0.0 – 19.7; 5.2)	0.20 (0.0 – 1.9; 1.9)	0.10 (0.0 – 24.7; 8.8)	2.10 (0.0 – 12.0; 7.4)	0.00 (0.0 – 2.8; 0.0)
%CD27+	43.6 (15.2–62.3; 27.9)	23.1 (9.4–60.4; 51.0)	50.2 (10.8–79.3; 40.5)	44.3 (25.6–72.4; 37.7)	NI^1^
**Concentration (pg/mL) of cytokines in plasma after stimulation with MABSC²**					
IFN-γ	227.5 (37–4268; 387.8)	102.0 (76–1890; 1302.0)	56.0 (1–1062; 205.0)	236.0 (1–2426; 797.0)	196.0 (7–799; 393.0)
TNF-α	950.5 (29–3864; 1125.0)	455.0 (132–2746; 2123.0)	310.0 (51–2213; 391.0)	569.0 (76–9169; 2725.0)	531.5 (70–1216; 678.0)
IL-2	121.0 (0–594; 239.5)	92.0 (26–236; 151.0)	56.0 (0–949; 184.0)	10.0 (0–123; 84.0)	0.5 (0–73; 45.0)
IL-17	13.5 (0–27; 14.0)	0.0 (0–19; 9.5)	0.0 (0–40; 3.0)	1.0 (0–41; 4.0)	0.0 (0–1; 1.0)
CD40L	120.0 (0–788; 165.8)	110.0 (24–531; 290.5)	7.0 (0–343; 96.0)	0.0 (0–137; 39.0)	0.0 (0–216; 56.0)
IL-4	0.0 (0–31; 12.0)	0.0 (0–35; 21.0)	0.0 (0–4; 0.0)	0.0 (0–35; 11.0)	0.0 (0–1; 0.0)
IL-5	1.5 (0–15; 2.8)	0.0 (0–2; 2.0)	2.0 (0–8; 3.0)	0.0 (0–8; 2.0)	0.0 (0–15; 2.0)
**Anti-MABSC IgG levels in plasma (OD)**	0.76 (0.27–3.42; 0.97)	0.52 (0.21–0.8; 0.33)	0.41 (0.13–2.6; 0.33)	0.31 (2.3–1.1; 0.11)	0.38 (0.19–0.59; 0.28)

^1^Not included (non-vaccinated controls were not included in these analyses, since only three of them had increased lymphoblast formation upon PBMC stimulation with MABSC).

^2^Net value, after subtracting the concentration of the cytokine in plasma of unstimulated whole blood.

### Assessment of the Cellular Immune Response to MABSC

With this assay, we aimed to analyse whether there is a T-B cell polarisation in response to MABSC. First, peripheral blood mononuclear cells (PBMC) were separated from whole blood, using a gradient density separation method, and frozen in liquid nitrogen (-196°C) until analysis. The PBMC separation, freezing and thawing procedures are better detailed in **Supplement 2**. After thawing, PBMC (5x10^5^ cells) were cultured for four days at 37°C with 5% CO_2_ in medium containing 80% of RPMI 1640 and 20% of foetal bovine serum in the presence of the mitogenic stimulant Phytohemagglutinin (PHA, 7.5 µg/mL, Sigma-Aldrich, used as a positive control) and an *in-house* MABSC lysate (10 µg/mL). The antigen preparation followed a previously described method (Closs et al., 1980; Qvist et al., 2015b) and is summarized in **Supplement 2**. RPMI medium alone was used as a negative control. Each culture set had a final volume of 1 mL in 12x75 mm polystyrene cytometry tubes. The four-day culture period was chosen after optimisation assays (data not shown).

After being cultured, the PBMC were stained with fluorophore-conjugated monoclonal antibodies (eBioscience), targeting the surface markers CD45 (PE), CD3 (FITC), CD4 (PE-Cy.7), CD8 (APC), CD19 (eFluor-450), CD45RO (APC-eFluor-780) and CD27 (SuperBright-702), and underwent flow cytometric analysis in an Attune NxT flow cytometer (Invitrogen). For each sample/culture condition, we counted 100,000 events, which were analysed using the FlowJo Software version 10.7.2 (FlowJo, LLC). Briefly, we selected all leukocytes (cells expressing the surface CD45 marker), among which we selected single cells expressing the CD3 (T cell) and CD19 (B cell) surface markers. Within each cell type, we determined the intensity of the anti-MABSC cellular response by calculating the rate of lymphoblast formation upon lymphocyte stimulation with MABSC, given by the percentage of lymphoblasts among the total cells (resting lymphocytes plus lymphoblasts). The method based on lymphoblast count was developed by Gaines et al. (1996) 26 years ago and we have successfully used it in a previous study (Mazzola et al., 2007). To ascertain its reliability, we assessed cell samples from 20 volunteers from another study of our group and measured the rate of activated cells (expressing the activation markers CD38 and HLA-DR) within resting lymphocytes and lymphoblasts formed upon stimulation with PHA. The rate of CD38+ HLA-DR+ cells was more than 20 times higher within lymphoblasts when compared to resting lymphocytes (47.30 vs. 1.96%, p < 0.001) (**Supplementary Figure 1**). The net result was calculated after subtracting the background lymphoblast formation from unstimulated cells. For graphical purposes, the lymphoblast rate (LR) values were plotted as their square roots. Within the CD3+ lymphoblasts, we determined the rate of lymphoblasts expressing the CD4 and CD8 surface markers, and the rate of cells expressing the memory T cell marker CD45RO within each of these subtypes. Within the CD19+ lymphoblasts, we determined the rate of cells expressing the CD27+ marker, present in plasmablasts and memory B cells. The cell staining method and the analysis strategy are detailed in **Supplementary Figure 2**.

### Measurement of Cytokine Production in Response to MABSC

We performed this assay to complement the cell-based flow cytometry assay and visualise a possible T-B cell polarisation in response to MABSC. We also aimed to simulate a QuantiFERON-like cytokine release assay (Hamada et al., 2021) and investigate its diagnostic potential. Heparinised whole blood samples were transferred to 12x75 mm polystyrene cytometry tubes and stimulated overnight with PHA at 7.5 µg/mL (positive control) and MABSC lysate at 10 µg/mL. Non-stimulated samples were used as negative controls. The final volume in each tube was 1 mL. The tubes were then spun at 1000 x g for 15 minutes, the supernatant plasma was separated and frozen at -80°C until analysis. The concentrations (pg/mL) of IFN-γ, TNF-α, soluble CD40 Ligand (CD40L), interleukin (IL-) 2, 4, 5 and 17 in plasma were measured using a customised 7-plex kit for Luminex^®^ (R&D Systems) in accordance with the manufacturer’s instructions. The assay was read using the Bio-Plex Multiplex Immunoassay System (Bio-Rad). To analyse the cytokine pattern in response to MABSC lysate, we combined the concentration values of different cytokines by adding them together and called them T helper (Th)1 (IFN-γ + TNF-α + IL-2), Th2 (CD40L + IL-4 + IL-5) and Th17 (IL-17) cytokine levels (Mousset et al., 2019; Díaz et al., 2021). The cytokine concentrations were firstly expressed in picograms per mL (pg/mL). The variation in the cytokine concentration was expressed as the result of the concentration in plasma from stimulated blood minus the concentration in plasma from non-stimulated blood. For graphical purposes, these results were plotted as their square roots.

### Measurement of Anti-MABSC IgG in Plasma

We performed an ELISA assay to investigate the role of specific IgG antibodies in latent MABSC infection. This method has been previously published (Qvist et al., 2015b). Briefly, a 96-well microtiter plate was covered with the above mentioned MABSC lysate (diluted 1/2000). After blocking nonspecific binding sites in the microtiter plate, the plasma samples (diluted 1/100) were added, along with eight dilutions (1/500 to 1/64000) of a pooled serum from known MABSC cases, which was used to generate a standard curve, the optical density (OD) values of which were converted to ELISA units per millilitre (U/mL). We used Receiving Operating Characteristics (ROC) analysis to define three cut-off values: 125, 125-400 and >400 U/mL, which indicated low, moderate, and high risk of MABSC infection, respectively (Qvist et al., 2015b). The results are routinely expressed as U/mL. However, for statistical and graphical purposes, we decided to rely on the OD values, since some concentration values were out of the standard curve (>640 U/mL), making them impossible to be precisely quantified by ELISA units. These concentration values are available in **Supplementary Table 3**.

### Hypotheses

In accordance with the cross-reactivity between mycobacterial species as mentioned above, we hypothesised: **a)** that lymphocytes from both CF patients with history of MABSC, and non-CF controls vaccinated with BCG would proliferate with similar intensity in response to MABSC, **b)** that these groups would have a more intense lymphocyte proliferation in comparison to the CF/NTM- and C/BCG- groups, **c)** that the rate of CD3+ cells would be higher than the rate of CD19+ cells in response to MABSC, **d)** with increased numbers of CD4+ cells in comparison with CD8+ cells, and **e)** with increased levels of Th1 cytokines in comparison with Th2 and Th17 cytokines. We also expected, as we have previously shown (Abate et al., 2019), that the specific anti-MABSC IgG levels would be significantly higher in CF patients with MABSC history than in all other groups.

### Statistical Analyses

For cell subtype analyses, we only included individuals who had increased CD3+ or CD19+ lymphoblast formation upon PBMC stimulation with MABSC. All numerical variables were expressed as the medians, followed by minimum, maximum values, and interquartile ranges (IQR). Since the transformed data did not achieve normal distribution, non-transformed values were used for the statistical tests. CF patients with MAC history were not included in the unpaired analyses due to the low number of individuals in this group. Differences between two different groups were assessed by the Mann-Whitney U test, with the Bonferroni correction being used when doing more than one unpaired comparison. Differences between related samples were assessed by the Wilcoxon W test (two samples) and by the Friedman test followed by the Dunn pairwise correction (more than two samples). For all tests, a *p*-value ≤ 0.05 indicated statistical significance. The statistical analyses were made using GraphPad Prism for Windows v. 8.4.3 (GraphPad Software).

## Results

### Cellular Immune Response to MABSC Was Seen in CF Patients With and Without History of MABSC Infection, With Predominant T Cell Activity

**Figure 1:**
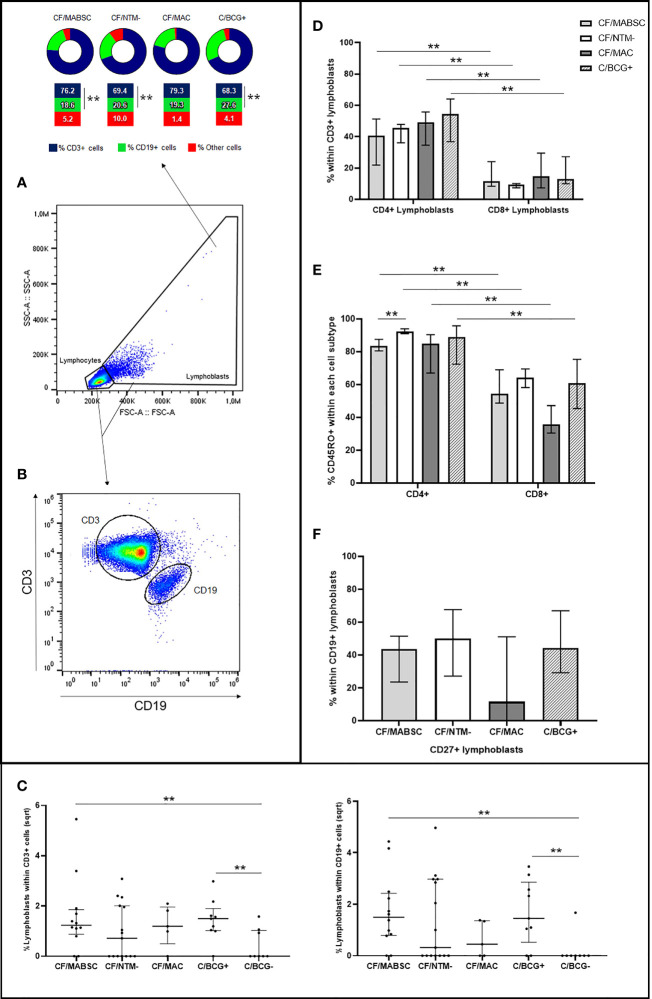
Graphs showing **(A)** the rate of CD3+ and CD19+ cells within the lymphoblasts formed upon PBMC stimulation with MABSC in CF patients with history of MABSC infection (CF/MABSC), CF patients without history of NTM infection (CF/NTM-), CF patients with history of MAC infection (CF/MAC), and non-CF controls vaccinated with BCG (C/BCG+). Non-vaccinated controls (C/BCG-) were not included in the cell subtype analyses due to the low number of individuals who had lymphoblast formation upon PBMC stimulation with MABSC; **(B)** Selection of CD3+ and CD19+ cells and **(C)** lymphoblast formation rate within CD3+ (to the left) and CD19+ cells (to the right) in the CF/MABSC, CF/MAC, CF/NTM-, C/BCG+ and C/BCG- groups. The results are shown as the square roots of the rates of lymphoblasts within the total cells (resting lymphocytes plus lymphoblasts, excluding background lymphoblast formation from non-stimulated cells). The bold middle lines in the plots indicate the medians and the lower and upper lines indicate the 25^th^ and 75^th^ percentiles, respectively. The extremities of the lines above the plots indicate differences between two groups with **p ≤ 0.01; **(D)** Rate of CD4+ and CD8+ cells within the CD3+ lymphoblasts formed upon PBMC stimulation with MABSC; **(E)** Rate of CD45RO+ cells within the CD4+ and CD8+ lymphoblasts; **(F)** Rate of CD27+ cells within CD19+ lymphoblasts formed upon PBMC stimulation with MABSC. In **(D–F)**, the extremities of the bars indicate the medians and the lower and upper lines in the bars indicate the 25^th^ and 75^th^ percentiles, respectively, while the extremities of the longer lines above the bars indicate differences between two related samples with the extremities of the shorter lines above the bars indicate difference between groups with **p ≤ 0.01.

All 12 (100%) individuals in the CF/MABSC group and 10/15 (67.7%) in the CF/NTM- group had increased CD3+ or CD19+ lymphoblast formation upon stimulation of their PBMC with MABSC lysate (**Table 1**). There was no significant difference in the proportion of individuals who had increased lymphoblast formation between the groups. In both groups, there was a significantly higher rate of CD3+ than CD19+ cells among the lymphoblasts (**Figure 1A**). The LRs within CD3 and CD19 cells were not significantly different between the groups (**Figures 1B, C**).

Within CD3+ lymphoblasts, there was a significantly higher rate of CD4+ than CD8+ cells in both groups, when compared to the CF/MABSC group (**Figure 1D**). Within CD4+ and CD8+ lymphoblasts, most cells expressed the memory T cell marker CD45RO in both groups. The rate of CD4+/CD45RO+ cells was significantly higher than the rate of CD8+/CD45RO+ cells in both groups, and the rate of CD4+/CD45RO+ cells was significantly higher in the CF/NTM- group, when compared to the CF/MABSC group (**Figure 1E**). Within CD19+ lymphoblasts, the median rate of cells expressing CD27 was 43.6% in the CF/MABSC group and 50.2% in the CF/NTM- group, without significant difference between them (**Table 1** and **Figure 1F**).

**Figure 2:**
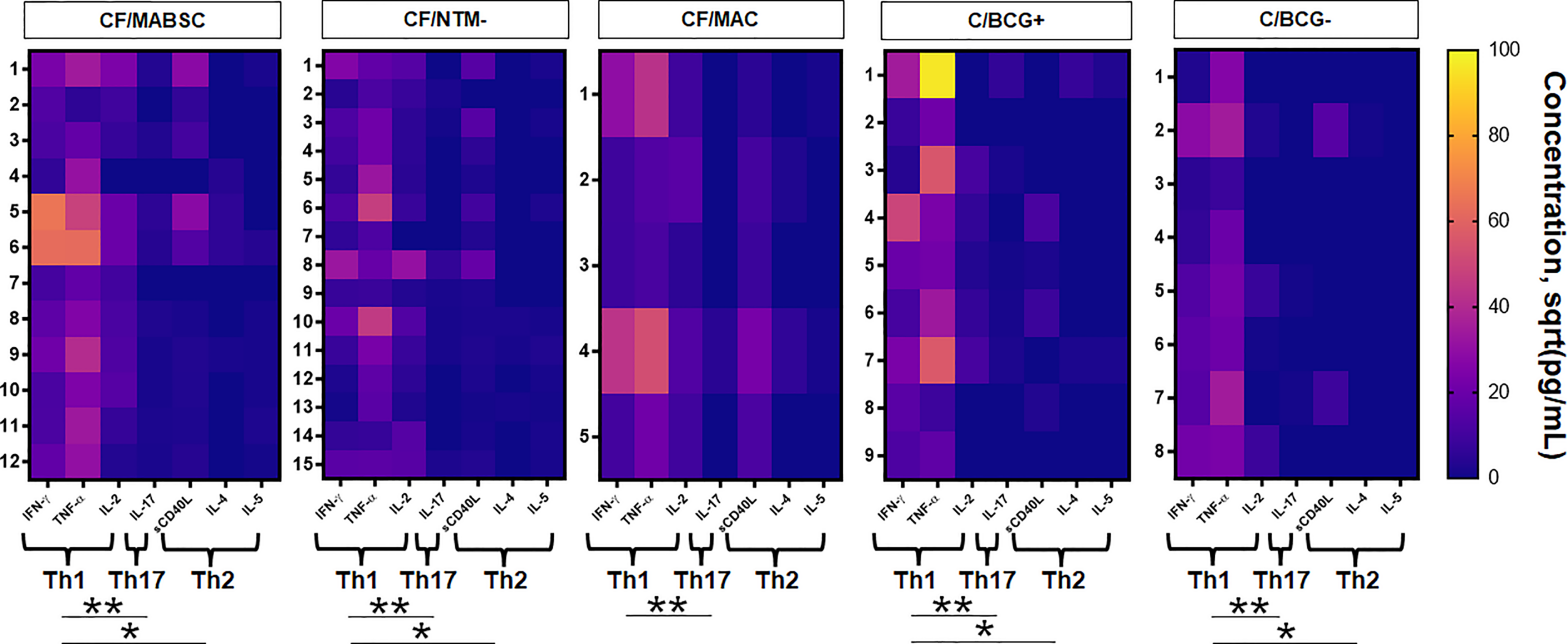
Heatmaps showing the activity of IFN-γ, TNF-α, IL-2, IL-17, soluble CD40L, IL-4 and IL-5 upon overnight whole blood stimulation with MABSC in the CF/MABSC, CF/MAC, CF/NTM-, C/BCG+ and C/BCG- groups. The results are expressed as the square roots of the concentrations of each cytokine. The braces below each cytokine set indicate the combined concentration values of each cytokine, forming different cytokine patterns – T helper (Th)1 (IFN-γ + TNF-α + IL-2), Th2 (IL-4 + IL-5 + CD40L) and Th17 (IL-17) cytokine levels. *p ≤ 0.05; **p ≤ 0.01.

There was a significant increase in the CD4 T cell-associated cytokines IFN-γ, TNF-α and IL-2 after overnight whole blood stimulation with MABSC in both groups, without difference between groups. The concentration of the B cell-associated cytokine soluble CD40L significantly increased upon stimulation with MABSC in both groups, without difference between them. No pre- and post-stimulation changes were seen for the B-cell associated cytokine IL-4. Discrete, yet significant increase was seen for IL-5 and IL-17 (**Supplementary Figure 3**). The combined Th1 cytokine level was significantly higher than the combined Th2 and the Th17 cytokine levels in the CF/MABSC and CF/NTM- groups after stimulation with MABSC. The CF/MABSC group had a significantly higher IL-2 concentration in plasma upon MABSC stimulation than the C/BCG+ and C/BCG- groups (**Figure 2**). The post-stimulation concentrations of all measured cytokines were not associated with duration of infection in patients with a history of MABSC infection (**Supplementary Table 4**).

### Increased Anti-MABSC IgG Levels in CF Patients but not in Controls

**Figure 3:**
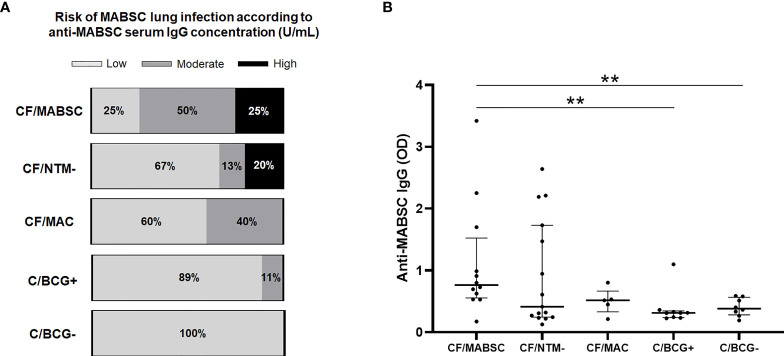
**(A)** Bar graphs showing the rate of patients in the CF/MABSC, CF/NTM-, CF/MAC, C/BCG+ and C/BCG- groups with anti-MABSC IgG levels associated with low (<125 U/mL), moderate (125-400 U/mL) and high risk (>400 U/mL) of MABSC infection, as previously reported (Qvist et al., 2015b). **(B)** Dot plots showing the plasma levels of anti-MABSC IgG, as indicated by optical density (OD) values, in the same groups. The bold middle lines in the plots indicate the medians and the lower and upper lines indicate the 25^th^ and 75^th^ percentiles, respectively. The extremities of the lines above the plots indicate differences between two groups with **p ≤ 0.01.

According to their anti-MABSC IgG concentration in plasma (median values shown in **Table 1**), 3/12 (25%) individuals in the CF/MABSC group had low risk, six (50%) had moderate risk, and three (25%) had high risk of MABSC infection. In the CF/MAC group, 3/5 (60%) individuals had low risk and 2/5 (40%) had moderate risk of MABSC infection. In the CF/NTM- group, 10/15 (67%) had low risk, 2/15 (13%) had moderate risk, and 3/15 (20%) had high risk of MABSC infection. In the C/BCG+ group, 8/9 (89%) individuals had low risk and 1/9 (11%) had moderate risk of MABSC infection. In the C/BCG- group, all eight individuals (100%) had low risk of MABSC infection (**Figure 3A**). The IgG levels were significantly higher in the CF/MABSC group when compared to the C/BCG+ and C/BCG- groups, but not the CF/NTM- group (**Figure 3B**). The plasma anti-MABSC IgG levels in patients with MABSC infection did not significantly differ according to the duration of infection (**Supplementary Table 4**).

### Cellular Immune Response to MABSC Seen in CF Patients With History of MAC Infection and BCG-Vaccinated Controls

Four out of five (80%) patients with MAC history and 8/9 (88.9%) BCG-vaccinated controls had increased CD3+ or CD19+ lymphoblast formation upon PBMC stimulation with MABSC (**Supplement 1**, **Supplementary Table 1**). The cellular immune response was similar in both groups and followed the pattern seen in the CF/MABSC and CF/NTM- groups. In the C/BCG+ group, there was a higher rate of CD3+ than CD19+ lymphoblasts, higher rate of CD4+ than CD8+ lymphoblasts and higher rate of CD4+/CD45RO+ lymphoblasts than CD8+/CD45RO+ lymphoblasts. Within the CD19+ lymphoblasts, the median rate of cells expressing CD27 was 44.3%. BCG-vaccinated controls also had a higher rate of CD19+ (B) cells among the total lymphoblasts formed upon PBMC stimulation with MABSC, when compared to CF patients with MABSC history and without NTM history (**Figure 1**).

The CF/MAC group tended to have an increase in the plasma concentration of IFN-γ and TNF-α upon overnight whole blood stimulation with MABSC, and no increase trend was seen for the other cytokines. In the C/BCG+ group, IFN-γ and TNF-α had a significant increase upon MABSC stimulation, but no differences were seen for the other cytokines (**Supplementary Figure 3**). Like in the CF/MABSC and CF/NTM- groups, the combined Th1 cytokine level was significantly higher than the combined Th2 and the Th17 cytokine levels upon stimulation with MABSC in the C/BCG+ group (**Figure 2**).

### Lymphoblast Formation and Increased IFN-γ and TNF-α Production in Controls Not Vaccinated With BCG

Three out of eight (37.5%) non-vaccinated controls (C/BCG- group) had increased CD3+ or CD19+ lymphoblast formation upon stimulation of their PBMC with MABSC (**Table 1**). Due to this low, statistically powerless number, we did not include these individuals in the cell subtype analyses. This group also had a significant increase in the plasma concentration of IFN-γ and TNF-α, but not the other cytokines, upon overnight whole blood stimulation with MABSC (**Supplementary Figure 3**). The combined Th1 cytokine level was significantly higher when compared to the combined Th2 and Th17 cytokine levels (**Figure 2**).

## Discussion

We found that the response to MABSC lung infection in CF follows a pattern seen in other mycobacterial infections, with a major role of CD4+ T cells, increased production of IFN-γ, TNF-α and IL-2, and low production of IL-5, meaning a Th1-dominated response (Bieri et al., 2016; Ellis et al., 2017; Marçal et al., 2020). The B cell response, with CD40L and IgG production, is also present, but is less pronounced. The same seems to be the case for CF patients with history of MAC infection, suggesting cross-reactive immune response, although statistical analyses were not possible for this group, due to the low number of individuals. Cross-reaction can also be suggested by the T and B cell responses of BCG-vaccinated non-CF controls, which had similar intensity, in terms of lymphocyte proliferation, when compared to CF patients with history of MABSC infection. However, they did not differ from patients without NTM history in this regard, also suggesting that CF patients without current NTM infection are exposed to or colonised with environmental NTM throughout life, which may prime their immune system.

### The Predominance of CD4 T Cells, With Increased Th1 Cytokine Production Indicates Response of the Th1 Type in the Lungs and Non-Disseminated Infection

The pivotal role of the Th1 response is best exemplified by the damaging clinical course of tuberculosis (TB) in the HIV epidemic, where low CD4 T cell counts predict progression from latent tuberculosis infection (LTBI) to active TB (Ellis et al., 2017), and also in *M. leprae* infection, where Th1/Th2 polarisation is crucial for the two clinical variants –paucibacillary tuberculoid leprosy and the multibacillary lepromatous leprosy (Pinheiro et al., 2018). In an animal study using a *Mycobacterium ulcerans* infection model, IFN-γ-deficient mice had a higher bacterial burden and accelerated disease progression (Bieri et al., 2016). Both IFN-γ and TNF-α production are hallmarks of the Th1 response and the production of both cytokines significantly increased in CF patients after whole blood stimulation with MABSC in the present study, indicating that the response to MABSC follows the same pattern seen in other mycobacterial infections, corroborating previous studies that showed increased production of these cytokines by human PBMC after challenge with MABSC (Sampaio et al., 2008; Jönsson et al., 2013; Steindor et al., 2015). The CD4 T cell activity against MABSC is also stressed by the increased production of IL-2, a classic marker of T cell activation, which induces both CD4 and CD8 T cells to differentiate into effector cells, while low IL-2 levels induce differentiation into follicular Th cells or central memory T cells. The strength and duration of the IL-2 signal also controls the expansion of CD8+ cells (Boyman and Sprent, 2012). Increased IL-2 production in CF patients (both with and without MABSC history), but not in non-CF controls, suggests that effector T cells are present in CF patients to some extent when their PBMC are challenged with MABSC and (in the case of patients without NTM history) possibly indicating previous exposure to NTM.

In case MABSC infection is present, the low (although significant) variation in the production of the Th17-associated cytokine IL-17 upon MABSC challenge suggest a low neutrophil recruitment, unlike what has been shown in *P. aeruginosa* lung infection in CF (Moser et al., 2000; Moser et al., 2005; Hartl et al., 2006). Also, the predominant Th1 response, with low IL-5 production, is in accordance with the TB paradigm of a non-disseminated infection (Furin et al., 2019). Notably, genetic impairment in the Th1 axis is associated with a higher risk of disseminated nontuberculous mycobacterial infection (Wu and Holland, 2015). A Th1-dominated response to *P. aeruginosa* in CF was also shown to be associated with improved outcome (Moser et al., 2000). Increased TNF-α production after stimulation with MABSC corroborates our statement, since, like IFN-γ in response to *M. tuberculosis*, *M. ulcerans* and *M. leprae* (Bieri et al., 2016; Ellis et al., 2017; Marçal et al., 2020), lower TNF-α levels are associated with a higher risk of disseminated infection. On the other hand, high TNF-α levels were shown to be associated with a higher risk of tissue injury (Stenger, 2005). Altogether, our findings suggest that the anti-MABSC response is Th1-dominated, not being necessarily suppressive or healing, with low risk of extrapulmonary disease. They also confirm that CF patients are not severely immunodeficient, even though some of them may have malnutrition, which is associated with reduced immune capacity (Elborn, 2016).

### The Humoral Immune Response Probably has a Secondary Role Against MABSC

In general, the role of the B cell response against mycobacteria is poorly explored. A recent review on TB proposes that the humoral response may help to control the disease severity and antibodies produced against *M. tuberculosis* have a good opsonisation capacity (Lewinsohn and Lewinsohn, 2019). In a MABSC setting, we have previously shown significantly increased serum IgG levels in CF patients with MABSC lung disease, indicating that they are rather a marker of clinical disease (Qvist et al., 2015b).

Although we have found that B cells also proliferate upon PBMC stimulation with MABSC, that plasmablasts are produced, as indicated by the presence of CD27+ cells, and that specific IgG is detectable in plasma of CF patients with and without MABSC history, the significantly lower rate of CD19+ compared with CD3+ cells among the total formed lymphoblasts suggests a less pronounced role of the B cell response. This is also indicated by the lower variation in the combined Th2 cytokine levels, associated with the antibody response (CD40L, IL-4 and IL-5) upon stimulation with MABSC, when compared to Th1-associated cytokines (IFN-γ, TNF-α and IL-2). Higher anti-MABSC IgG levels in CF patients with history of MABSC infection than in controls support the role of antibodies as biomarkers of active or past disease, but their protective capacity is less likely, considering the biofilm nature of MABSC lung infection (Qvist et al., 2015c), which is also the case for chronic *P. aeruginosa* biofilm infection (Bjarnsholt et al., 2009). For now, we show that B cells play a role in the anti-MABSC response, as they proliferate, interact with T cells, and produce antibodies when challenged with MABSC, but according to our cytokine results this role may be secondary when compared to T cells.

### Memory T and B Cells Are Formed in the Anti-MABSC Response

The presence of CD45RO+ T cells among both CD4+ and CD8+ lymphoblasts, and the presence of CD19+CD27+ cells indicate that memory cells are formed after PBMC are challenged with MABSC. CF patients without NTM history had a significantly higher rate of CD4+/CD45RO+ cells than patients in the CF/MABSC group (**Figure 1E**), which we believe is a result of selection bias, as we only selected individuals who had CD3+ lymphoblast formation for these analyses, ruling out almost half of the individuals in the CF/NTM- group. As these are ubiquitous bacteria in the environment (Bouso et al., 2017), the presence of memory CD4 and CD8 cells may indicate previous exposure in the form of subclinical infection or intermittent colonisation in the damaged lungs. We could not investigate past infections since systematic monthly NTM culture at our centre only began in 2012, and previous examinations were routinely made once a year or if clinicians suspected of NTM infection.

### Can BCG Vaccination Provide a Cross-Reactive Immune Response Against MABSC?

The internal genetic relatedness of mycobacteria (Pereira et al., 2020), like other bacterial genera (Høiby et al., 1987) is the reason for the cross-reactivity seen in the present study, which has been previously documented at our centre using both skin-based and serological tests (Hjelt et al., 1994; Qvist et al., 2015b). Our results are therefore in agreement with our hypothesis that non-CF individuals who were vaccinated with BCG have similar T- and B-cell proliferation intensity upon stimulation with MABSC when compared to CF patients with history of MABSC, both being significantly higher than non-vaccinated controls, indicating that BCG vaccination can provide a cross-reactive immune response against NTM. We corroborate the findings by Abate *et al.* – the first group to demonstrate such event. These authors found that PBMC from individuals with LTBI contained T cells that reacted against MABSC and MAC, and PBMC from BCG-vaccinated individuals were able to intracellularly inhibit these bacteria (Abate et al., 2019). Unexpectedly, in our study, BCG-vaccinated controls had a higher rate of CD19+ (B) cells among the total lymphoblasts formed upon PBMC stimulation with MABSC, when compared to CF patients with and without NTM history. Although this may be an effect of selection bias, we speculate that the better general health status of the controls compared to CF patients may account for this difference. Our finding that BCG-vaccinated controls also had similar plasma concentrations of IFN-γ and TNF-α upon MABSC stimulation when compared to patients with MABSC history suggests that a cross-reactive response provided by BCG may also act at the cytokine level. However, due to selection bias, our results should be interpreted carefully and a more rigorous investigation about the BCG effect is needed.

As mentioned, mycobacteria share a number of antigens that may trigger both T and B cell responses (Checkley et al., 2011; Qvist et al., 2015b; Qvist et al., 2017; Ravnholt et al., 2019), which has also been shown in a recent study using four MABSC antigens and two recombinant proteins (Le Moigne et al., 2021). As our *in-house* MABSC lysate is produced by means of X-press and sonication (Closs et al., 1980), it contains common mycobacterial antigens (Høiby et al., 1987; Qvist et al., 2015b), allowing us to measure a broader immune response to environmental NTM. The cross-reactive response may also be a result of trained immunity, that is, the nonspecific boosting of immune responses, in which BCG has been suggested to play a role (Koeken et al., 2021). On the other hand, there were no differences in the levels of any assessed cytokine in plasma from stimulated blood between vaccinated and non-vaccinated controls. Also, unlike CF patients, non-CF controls did not have a pronounced IL-2 production upon stimulation with MABSC, indicating that IL-2 may be an intrinsic player of the specific anti-MABSC or anti-NTM response.

### A Cross-Reactive Immune Response Yielded by BCG Is Not Necessarily Protective Against MABSC Infection

Most of the CF patients in our study who were vaccinated with BCG developed MABSC or MAC infection, although one of them was infected before being vaccinated. Another study found a 22.5% rate of NTM infection among 186 CF patients, all of whom were vaccinated with BCG (Ferroni et al., 2005). A smaller rate was shown by us in a similar cohort at the Campinas CF Centre, in Brazil (Aiello et al., 2018). This is not surprising, as, even in the TB setting, it is known that the BCG vaccine is poorly protective against latent infection (Dockrell and Smith, 2017). On the other hand, four out of five BCG-vaccinated CF patients in our study were able to clear NTM infection quickly. This suggests that, although the BCG vaccine is not necessarily protective against NTM infection as a whole, it may boost the immune response against clinical NTM infection, leading to a quicker resolution with decreased pathogenicity. Our thoughts are in accordance with a study in Uganda that assessed a cohort of neonates who randomly received the BCG vaccination at birth or six weeks after birth. A lower rate of diagnosed NTM infections was seen in the group who received BCG at birth, but the effect was lost during the follow-up after BCG was given six weeks after birth to the second group. The authors suggest that prioritising BCG vaccination at birth in settings with high mortality due to TB might have significant public health benefits through reductions in all-cause infectious morbidity and mortality (Prentice et al., 2021). At the moment, although our evidence is anecdotal, we think that the BCG vaccination should be assessed in the CF context as a potential tool for preventing NTM infection or at least reducing its pathogenicity. Still, we stress that more sophisticated studies are needed and the conclusions about the potential BCG benefits should be carefully drawn.

### The Potential of the Cross-Reactive Immune Response for Diagnosis of Latent or Subclinical Infection With MABSC and Other NTM

The Mantoux skin reaction, using tuberculin as antigen, cannot differentiate between *M. tuberculosis* infection and BCG vaccination due to cross-reactivity, for which reason the QuantiFERON TB test or Mantoux-like skin tests using *M. tuberculosis*-specific antigens are currently used for diagnosis (Hamada et al., 2021). Ideally, a similar approach would be preferable for diagnosing MABSC and other NTM in CF patients. Until that happens, however, cross-reactive responses among NTM may be used to diagnose latent or subclinical NTM infection in CF patients where the bacteria cannot be easily found and identified by routine culture- or molecular-based methods (Hjelt et al., 1994; Qvist et al., 2015a; Qvist et al., 2015b; Ravnholt et al., 2019). Our plan is, therefore, to carry out a prospective study to explore whether measuring both anti-MABSC IgG and the production of MABSC-lysate-induced IFN-γ (and possibly also TNF-α and IL-2) by blood cells will be useful for early diagnosis of NTM infections. We will especially focus on patients with possible latent or subclinical infection (Ho et al., 2021), and correlate our immunological tests with the monthly respiratory mycobacterial cultures we have been doing since 2012, lung function tests and chest high-resolution computed tomography. This may provide a better differentiation between colonisation, latent, subclinical, and clinically active infection, and also guide more invasive approaches, such as bronchoalveolar lavage, in order to accurately detect a specific NTM pathogen. Since the antibiotic treatment for NTM (especially MABSC) is difficult and has many side-effects (Desai and Hurtado, 2021), it would be tempting to try a shorter oral regimen in order to eradicate latent or subclinical NTM infection, as has been successfully done in TB (Dorman et al., 2021; Nordholm and Lillebaek, 2021). More accurate diagnosis may be obtained by assessing the response to purified, species-specific antigens, e.g., purified protein derivates (PPDs), from different NTM-species. However, such antigens have not been identified since purified tuberculin-like antigens (**“**sensitins**”**) from other NTM were unfortunately cross-reactive and not specific to a single NTM species (Hjelt et al., 1994).

### Study Limitations

The main limitation of our study was the low number of patients, which prevented us from doing further and more robust sub-group analyses. Since NTM prevalence and incidence are generally low in CF centres, multicentre studies are necessary for obtaining enough patients, especially for subgroup analyses – for example, differences in the patterns of immune response in CF patients with chronic MABSC infection and patients in whom the infection was successfully treated. Low number of individuals was also an issue regarding the non-CF control groups. We also did not include antigens of MAC, *M. tuberculosis* and BCG for this study, which would have allowed a more comprehensive evaluation of cross-reactivity.

Finally, we acknowledge the limitations of the lymphoblast counting technique to assess the intensity of the cellular response due to its low specificity. More sophisticated assays using a species-specific antigenic panel, a broader range of cell surface and intracellular cytokine makers, as well as assays based on gene expression for cytokine analysis, will be the focus of future studies.

## Conclusion

We show that the response to MABSC in CF follows a pattern seen in other mycobacterial infections, with a Th1-dominated response, characterised by the predominance of CD4 T cells, mainly coordinated by IFN-γ and TNF-α, and a secondary role of B cells, which contrasts with the Th2-skewed response seen in chronic *P. aeruginosa* infection. IL-2 increased in plasma of CF patients upon stimulation with MABSC, including in those without history of MABSC infection, and can be a cytokine involved in the specific anti-MABSC or anti-NTM response. It is unknown why CF patients without NTM history also showed reactivity, but previous colonisation or latent/subclinical infection leading to cross-reactive immune stimulation is a possible explanation. Likewise, we also demonstrate that non-CF controls vaccinated with BCG react to MABSC stimulation in a similar way as CF patients with history of MABSC infection, both in terms of lymphocyte activity, IFN-γ and TNF-α production.

Although our assays had low species-specificity due to cross reactions, we think that cross-reactivity can be used for genus-based diagnosis of NTM infections in the absence of more, species-specific tests. In that sense, we aim to evaluate the combination of the anti-MABSC IgG ELISA test and a cytokine release test for diagnosis of MABSC and other NTM in our CF patients prospectively. Finally, the usefulness of BCG vaccination to prevent or reduce the pathogenicity of NTM infections should be investigated in larger CF cohorts.

## Data Availability Statement

The original contributions presented in the study are included in the article/**Supplementary Material**. Further inquiries can be directed to the corresponding author.

## Ethics Statement

The study was approved by the Regional Scientific Ethical Committees, Denmark (VEK, RH-19022288) and was in accordance with the Declaration of Helsinki and guidelines from the Danish Data Protection Agency. All participants signed a written informed consent, confirming their voluntary participations.

## The Copenhagen Study Group on Mycibacterial Infections in Cystic Fibrosis

The following Authors, who are listed in alphabetical order, contributed to the work of the Copenhagen Study Group On Mycobacterial Infections In Cystic Fibrosis: Daniel Faurholt-Jepsen, Marco Gelpi, Neval Ete Wareham and Terese Katzenstein.

## Author Contributions

RMM: Conceptualization, data curation, formal analysis, investigation, methodology, administration, writing PØJ: methodology, administration, validation, visualization TQ: data curation, methodology, investigation, validation, visualization, resources MK: investigation, ethics, resources, validation, visualization CM: validation, visualization, funding acquisition TP: validation, visualization, resources MTNS: Conceptualization, data curation, formal analysis, investigation, methodology, administration, writing. NH: Initiator, conceptualization, analysis, methodology, writing. The Copenhagen Study Group on Mycobacterial infections in Cystic Fibrosis: patient recruitment, data collection, critical review. All authors contributed to the article and approved the submitted version.

## Funding

This study was funded by the São Paulo Research Foundation (FAPESP, grant no. 2019/14134-5 and 2019/08598-9) and by the Cystisk Fibrose Foreningen (Danish Cystic Fibrosis Association).

## Conflict of Interest

The authors declare that the research was conducted in the absence of any commercial or financial relationships that could be construed as a potential conflict of interest.

## Publisher’s Note

All claims expressed in this article are solely those of the authors and do not necessarily represent those of their affiliated organizations, or those of the publisher, the editors and the reviewers. Any product that may be evaluated in this article, or claim that may be made by its manufacturer, is not guaranteed or endorsed by the publisher.
