# Supplementary material for: Adaptive Immune Response to Mycobacterium abscessus Complex (MABSC) in Cystic Fibrosis and the Implications of Cross-Reactivity
Source: Front Cell Infect Microbiol. 2022 Apr 20;12:858398. doi: 10.3389/fcimb.2022.858398 (PMC9084186; doi:10.3389/fcimb.2022.858398)
Supplement: Supplementary file 1 [file DataSheet_1.docx]

**Supplement 1.** Characteristics of patients with cystic fibrosis (CF) and controls included in the study.

**Supplementary Table 1**. CF Patients with history of MABSC infection (highlighted in blue), history of MAC infection (highlighted in yellow) and without history of NTM infection (not highlighted). Red fonts indicate patients who were vaccinated with BCG. Below the bold border are the BCG-vaccinated controls (red font) and non-vaccinated controls (black font). Lymphoblast formation within CD3+ and CD19+ cells before (NS) and after (MABSC) PBMC stimulation with MABSC lysate.

| **IND** | **NTM history** | **BCG** | **CD3**  **MABSC** | **CD3 NS** | **CD3 final** |  | **CD19 MABSC** | **CD19 NS** | **CD19 final** |
| --- | --- | --- | --- | --- | --- | --- | --- | --- | --- |
| CFP001 | Yes | Yes | 7.39 | 4.08 | 3.31 |  | 14.90 | 14,7 | 0.20 |
| CFP002 | Yes | No | 3.46 | 2.82 | 0.64 |  | 20.06 | 15,08 | 4.98 |
| CFP003 | Yes | Yes | 2.87 | 1.57 | 1.30 |  | 34.70 | 28,5 | 6.20 |
| CFP004 | No | No | 12.80 | 3.30 | 9.50 |  | 14.10 | 5,24 | 8.86 |
| CFP005 | No | No | 2.48 | 1.38 | 1.10 |  | 16.70 | 17 | 0.00 |
| CFP006 | No | No | 1.60 | 1.74 | 0.00 |  | 25.70 | 28,3 | 0.00 |
| CFP007 | Yes | No | 3.69 | 1.75 | 1.94 |  | 15.70 | 13,1 | 2.60 |
| CFP008 | No | Yes | 9.14 | 3.35 | 5.79 |  | 28.90 | 20,04 | 8.86 |
| CFP009 | No | No | 2.31 | 1.44 | 0.87 |  | 10.06 | 14,6 | 0.00 |
| CFP010 | No | No | 6.81 | 2.96 | 3.85 |  | 20.03 | 10,3 | 9.73 |
| CFP011 | No | No | 7.02 | 1.50 | 5.52 |  | 22.50 | 14,4 | 8.10 |
| CFP012 | Yes | Yes | 2.84 | 1.86 | 0.98 |  | 16.10 | 14,2 | 1.90 |
| CFP013 | Yes | No | 2.67 | 2.99 | 0,00 |  | 15.30 | 13,5 | 1.80 |
| CFP014 | No | No | 1.69 | 1.79 | 0.00 |  | 22.10 | 28,6 | 0.00 |
| CFP015 | Yes | No | 7.30 | 3.66 | 3.64 |  | 21.08 | 21,1 | 0.00 |
| CFP016 | Yes | No | 6.08 | 4.34 | 1.74 |  | 15.00 | 24,7 | 0.00 |
| CFP017 | Yes | No | 7.22 | 2.84 | 4.38 |  | 13.10 | 14,2 | 0.00 |
| CFP018 | Yes | No | 6.91 | 4.05 | 2.86 |  | 22.10 | 20,2 | 1.90 |
| CFP019 | Yes | No | 3.92 | 2.50 | 1.42 |  | 15.00 | 16,6 | 0.00 |
| CFP020 | Yes | Yes | 4.00 | 2.68 | 1.32 |  | 24.00 | 21,8 | 3.00 |
| CFP021 | Yes | Yes | 2.68 | 3.07 | 0.39 |  | 21.50 | 20,8 | 0.70 |
| CFP022 | Yes | No | 2.03 | 2.48 | 0.45 |  | 9.78 | 8,82 | 0.96 |
| CFP023 | Yes | No | 13.3 | 1.79 | 11.51 |  | 31.70 | 14,3 | 17.40 |
| CFP024 | Yes | No | 33.9 | 4.10 | 29.80 |  | 36.20 | 16,5 | 19.70 |
| CFP025 | No | No | 7.33 | 3.30 | 4.03 |  | 35.30 | 10,6 | 24.70 |
| CFP026 | No | No | 3.31 | 4.06 | 0.00 |  | 18.50 | 18,6 | 0.00 |
| CFP027 | No | No | 2.75 | 3.70 | 0.00 |  | 20.10 | 20 | 0.10 |
| CFP028 | No | No | 3.15 | 4.86 | 0.00 |  | 24.50 | 23,5 | 1.00 |
| CFP029 | No | No | 1.98 | 2.19 | 0.00 |  | 15.50 | 15,5 | 0.00 |
| CFP030 | Yes | No | 6.91 | 5.68 | 1.23 |  | 20.30 | 19,7 | 0.60 |
| CFP031 | No | No | 3.74 | 3.23 | 0.51 |  | 23.30 | 18,8 | 4.20 |
| CFP032 | No | No | 2.06 | 2.86 | 0.00 |  | 21.10 | 21,1 | 0.00 |
|  |  |  |  |  |  |  |  |  |  |
| NC001 | No | Yes | 5.57 | 3.33 | 2.24 |  | 31.50 | 19,5 | 12.00 |
| NC002 | No | Yes | 2.74 | 1.74 | 1.00 |  | 23.50 | 18,1 | 5.40 |
| NC003 | No | No | 3.80 | 2.68 | 1.12 |  | 27.80 | 29,4 | 0.00 |
| NC004 | No | Yes | 3.56 | 2.48 | 1.08 |  | 21.60 | 19,5 | 2.10 |
| NC005 | No | Yes | 3.51 | 5.51 | 0.00 |  | 27.10 | 27,3 | 0.00 |
| NC006 | No | No | 2.42 | 3.42 | 0.00 |  | 26.80 | 30,8 | 0.00 |
| NC007 | No | No | 1.82 | 2.46 | 0.00 |  | 19.50 | 20,8 | 0.00 |
| NC008 | No | No | 1.68 | 1.95 | 0.00 |  | 22.60 | 24,7 | 0.00 |
| NC009 | No | Yes | 3.23 | 1.81 | 1.42 |  | 28.00 | 21,4 | 6.60 |
| NC010 | No | No | 3.43 | 4.38 | 0.00 |  | 28.90 | 29 | 0.00 |
| NC011 | No | No | 2,35 | 2.42 | 0.00 |  | 25.40 | 25,9 | 0.00 |
| NC012 | No | Yes | 7,26 | 2.51 | 4.75 |  | 23.30 | 22,2 | 1.10 |
| NC013 | No | Yes | 7,63 | 5.18 | 2.45 |  | 17.50 | 16,3 | 1.20 |
| NC014 | No | No | 5,76 | 4.89 | 0.87 |  | 27.80 | 25 | 2.80 |
| NC015 | No | Yes | 20,7 | 11.80 | 8.90 |  | 33.20 | 23,3 | 9.90 |
| NC016 | No | No | 17,2 | 14.70 | 2.50 |  | 22.20 | 22,9 | 0.00 |
| NC017 | No | Yes | 13,2 | 10.60 | 2.60 |  | 35.00 | 34,3 | 0.00 |

^Note: Negative values are expressed as zero.^

**Supplementary Table 2**. Characteristics of CF patients enrolled in the study. Age, gender, history of MABSC infection, duration of MABSC infection, number of positive cultures and fulfillment of the ATS/IDSA criteria.

| **Individual** | **Age** | **Gender** | **History of NTM infection in microbiological** | **Number of** | **Fulfilled** |
| --- | --- | --- | --- | --- | --- |
|  |  |  | **culture of respiratory specimens** | **positive cultures** | **ATS/IDSA criteria** |
| CFP001 | 22.3 | Male | *M. chimaera* since 2016 | 6 | Yes |
| CFP002 | 27.1 | Female | MABSC from 2014 to 2015 | 8 | Yes |
| CFP003 | 21.9 | Female | MABSC from 2009 to 2016 | 63 | Yes |
| CFP004 | 27.9 | Male | - |  | - |
| CFP005 | 21.6 | Male | - |  | - |
| CFP006 | 35.7 | Male | - |  | - |
| CFP007 | 21.4 | Female | MABSC in 2019 | 2 | Yes |
| CFP008 | 21.3 | Female | - |  | - |
| CFP009 | 24.1 | Male | - |  | - |
| CFP010 | 21.4 | Female | - |  | - |
| CFP011 | 22.1 | Female | - |  | - |
| CFP012 | 26.1 | Female | MAC in 2019 | 2 | Yes |
| CFP013 | 30.5 | Male | MAC from 2005 to 2009 | 7 | Yes |
| CFP014 | 37.9 | Female | - |  | - |
| CFP015 | 19.6 | Male | MABSC since 2013 | 54 | Yes |
| CFP016 | 31.4 | Male | MABSC in 2013 | 6 | Yes |
| CFP017 | 24.0 | Male | MAC since 2011 | 40 | Yes |
| CFP018 | 24.7 | Male | MABSC since 2012 | 15 | Yes |
| CFP019 | 40.0 | Female | MAC (2010-2012, 2014 and 2017) | 15 | Yes |
| CFP020 | 28.6 | Female | MABSC from 2012 to 2013 | 5 | Yes |
| CFP021 | 50.6 | Female | MABSC in 2000 and *M. fortuitum* in 2016^ǂ^ | 1 each | Yes |
| CFP022 | 30.9 | Male | MABSC from 2011 to 2017 | 43 | Yes |
| CFP023 | 34.8 | Male | MABSC* since 2016 and *M. chimaera*** since 2018 | *11 and **5 | Yes |
| CFP024 | 21.4 | Male | MABSC in 2015 | 5 | Yes |
| CFP025 | 45.2 | Female | - |  | - |
| CFP026 | 41.0 | Female | - |  | - |
| CFP027 | 21.6 | Female | - |  | - |
| CFP028 | 29.4 | Female | - |  | - |
| CFP029 | 56.2 | Male | - |  | - |
| CFP030 | 36.4 | Female | MABSC from 2012 to 2016 | 12 | Yes |
| CFP031 | 26.6 | Male | - |  | - |
| CFP032 | 34.9 | Female | - |  | - |

**Supplementary Table 3.** Anti-MABSC IgG measurement in plasma samples of CF patients and non-CF controls. Results given as optical density (OD) and concentration in units per mililiter (U/mL).

| **CF patients** | | | |  | **Non-CF controls** | | | |
| --- | --- | --- | --- | --- | --- | --- | --- | --- |
| **IND** | **NTM history** | **OD** | **U/mL** |  | **IND** | **BCG** | **OD** | **U/mL** |
| CFP001 | Yes | 0.449 | 82.5 |  | NC001 | Yes | 0.243 | 10.7 |
| CFP002 | Yes | 1.700 | 467.0 |  | NC002 | Yes | 0.310 | 19.9 |
| CFP003 | Yes | 0.729 | 151.0 |  | NC003 | No | 0.265 | 12.2 |
| CFP004 | No | 0.610 | 121.0 |  | NC004 | Yes | 1.100 | 194.0 |
| CFP005 | No | 2.210 | >640.0 |  | NC005 | Yes | 0.226 | 9.4 |
| CFP006 | No | 0.946 | 209.0 |  | NC006 | No | 0.580 | 77.6 |
| CFP007 | Yes | 0.531 | 102.0 |  | NC007 | No | 0.401 | 33.7 |
| CFP008 | No | 0.414 | 74.1 |  | NC008 | No | 0.365 | 23.4 |
| CFP009 | No | 0.231 | 31.7 |  | NC009 | Yes | 0.313 | 19.9 |
| CFP010 | No | 0.270 | 40.0 |  | NC010 | No | 0.191 | 7.9 |
| CFP011 | No | 0.308 | 48.7 |  | NC011 | No | 0.333 | 17.9 |
| CFP012 | Yes | 0.214 | 28.3 |  | NC012 | Yes | 0.361 | 22.5 |
| CFP013 | Yes | 0.531 | 102.0 |  | NC013 | Yes | 0.315 | 16.2 |
| CFP014 | No | 2.190 | >640.0 |  | NC014 | No | 0.513 | 61.7 |
| CFP015 | Yes | 3.420 | >640.0 |  | NC015 | Yes | 0.231 | 9.8 |
| CFP016 | Yes | 0.910 | 199.0 |  | NC016 | No | 0.588 | 79.5 |
| CFP017 | Yes | 0.801 | 170.0 |  | NC017 | Yes | 0.330 | 17.5 |
| CFP018 | Yes | 0.530 | 102.0 |  |  |  |  |  |
| CFP019 | Yes | 0.516 | 98.4 |  |  |  |  |  |
| CFP020 | Yes | 0.625 | 125.0 |  |  |  |  |  |
| CFP021 | Yes | 0.173 | 20.0 |  |  |  |  |  |
| CFP022 | Yes | 2.250 | >640.0 |  |  |  |  |  |
| CFP023 | Yes | 0.697 | 143.0 |  |  |  |  |  |
| CFP024 | Yes | 0.988 | 221.0 |  |  |  |  |  |
| CFP025 | No | 0.125 | 8.34 |  |  |  |  |  |
| CFP026 | No | 0.320 | 51.4 |  |  |  |  |  |
| CFP027 | No | 0.218 | 29.1 |  |  |  |  |  |
| CFP028 | No | 2.640 | >640.0 |  |  |  |  |  |
| CFP029 | No | 1.730 | 478.0 |  |  |  |  |  |
| CFP030 | Yes | 0.799 | 169.0 |  |  |  |  |  |
| CFP031 | No | 0.244 | 34.4 |  |  |  |  |  |
| CFP032 | No | 1.470 | 377.0 |  |  |  |  |  |

**Supplementary Table 4**. Lymphoblast formation rate within **(A)** CD3+ cells and **(B)** CD19+ cells, and **(C)** levels of anti-MABSC IgG in plasma in CF patients with history of NTM infection according to the infection duration

| A) CD3+ Lymphoblasts |  |  |  |  |
| --- | --- | --- | --- | --- |
| **Infection duration** | **One year** | **2-5 years** | **>Five years** | **p-value** |
| LP intensity | *(n = 8)* | *(n = 4)* | *(n = 5)* |  |
| **Min** | 0.00 | 0.00 | 0.00 |  |
| **MLR** | 1.53 | 1.33 | 2.86 | *ns* |
| **Max** | 4.38 | 11.51 | 3.64 |  |
| **IQR** | 2.78 | 8.68 | 2.83 |  |
|  |  |  |  |  |
| \| B) CD19+ Lymphoblasts \|  \|  \|  \|  \| \| --- \| --- \| --- \| --- \| --- \| \| **Infection duration** \| **One year** \| **2-5 years** \| **>Five years** \| **p-value** \| \| LP intensity \| *(n = 8)* \| *(n = 4)* \| *(n = 5)* \| \| **Min** \| 0.00 \| 0.00 \| 0.00 \|  \| \| **MLR** \| 2.25 \| 1.20 \| 0.96 \| *ns* \| \| **Max** \| 19.7 \| 17.4 \| 3.2 \|  \| \| **IQR** \| 4.31 \| 13.4 \| 3.6 \|  \| | | | | |
| \| MRL: Median Lymphoblast Rate (Stimulated minus unstimulated PBMC); ns: not significant \| \| \| \| --- \| --- \| --- \| \|  \|  \|  \| | | | | |
|  | | | | |
| C) Anti-MABSC IgG levels in plasma | | | | |
| **Infection duration** | **One year** | **2-5 years** | **>Five years** | **p-value** |
| IgG levels* | *(n = 8)* | *(n = 5)* | *(n = 5)* |  |
| **Min** | 0.173 | 0.449 | 0.530 |  |
| **Median OD** | 0.625 | 0.531 | 0.729 | *ns* |
| **Max** | 1.700 | 0.599 | 2.250 |  |
| **IQR** | 0.77 | 0.27 | 1.0 |  |
| *Given in optical density (OD); ns: not significant | |  |  |  |

**Supplemental Table 4.** Plasma concentrations of IFN-γ, TNF-α, IL-2, IL-17, CD40L, IL-4 and IL-5 in CF patients with history of NTM infection according to the infection duration.

| **Infection duration** | **One year** | **2-5 years** | **>5 years** | **p-value** |
| --- | --- | --- | --- | --- |
| IFN-γ concentration* | *(n = 8)* | *(n = 5)* | *(n = 5)* |  |
| **Min** | 78 | 76 | 37 |  |
| **Median concentration** | 146 | 139 | 433 | *ns* |
| **Max** | 4268 | 898 | 3891 |  |
| **IQR** | 443 | 496 | 2783 |  |
|  | | | | |
|  |  |  |  |  |
| **Infection duration** | **One year** | **2-5 years** | **>5 years** | **p-value** |
| IL-17 concentration* | *(n = 8)* | *(n = 5)* | *(n = 5)* |  |
| **Min** | 0 | 0 | 0 |  |
| **Median concentration** | 7 | 0 | 2 | *ns* |
| **Max** | 27 | 2 | 19 |  |
| **IQR** | 11 | 2 | 17 |  |
|  | | | | |
|  |  |  |  |  |
| **Infection duration** | **One year** | **2-5 years** | **>5 years** | **p-value** |
| TNF-α concentration* | *(n = 8)* | *(n = 5)* | *(n = 5)* |  |
| **Min** | 190 | 132 | 29 |  |
| **Median concentration** | 652 | 627 | 1639 | *ns* |
| **Max** | 2309 | 1821 | 3864 |  |
| **IQR** | 880 | 1137 | 2806 |  |
|  | | | | |
|  |  |  |  |  |
| **Infection duration** | **One year** | **2-5 years** | **>5 years** | **p-value** |
| CD40L concentration* | *(n = 8)* | *(n = 5)* | *(n = 5)* |  |
| **Min** | 0 | 3 | 0 |  |
| **Median concentration** | 108 | 24 | 41 | *ns* |
| **Max** | 788 | 145 | 531 |  |
| **IQR** | 773 | 102 | 355 |  |
|  |  |  |  |  |
|  |  |  |  |  |
| **Infection duration** | **One year** | **2-5 years** | **>5 years** | **p-value** |
| IL-2 concentration* | *(n = 8)* | *(n = 5)* | *(n = 5)* |  |
| **Min** | 48 | 26 | 0 |  |
| **Median concentration** | 142 | 88 | 170 | *ns* |
| **Max** | 594 | 222 | 389 |  |
| **IQR** | 338 | 113 | 240 |  |
|  |  |  |  |  |
|  |  |  |  |  |
| **Infection duration** | **One year** | **2-5 years** | **>5 years** | **p-value** |
| IL-4 concentration* | *(n = 8)* | *(n = 5)* | *(n = 5)* |  |
| **Min** | 0 | 0 | 0 |  |
| **Median concentration** | 0 | 0 | 15 | *ns* |
| **Max** | 31 | 2 | 35 |  |
| **IQR** | 7 | 1 | 32 |  |
|  | | | | |
|  |  |  |  |  |
| **Infection duration** | **One year** | **2-5 years** | **>5 years** | **p-value** |
| IL-5 concentration* | *(n = 8)* | *(n = 5)* | *(n = 5)* |  |
| **Min** | 0 | 0 | 0 |  |
| **Median concentration** | 0 | 0 | 2 | *ns* |
| **Max** | 6 | 2 | 15 |  |
| **IQR** | 3 | 2 | 9 |  |
