# Supplementary material for: Adaptive Immune Response to Mycobacterium abscessus Complex (MABSC) in Cystic Fibrosis and the Implications of Cross-Reactivity
Source: Front Cell Infect Microbiol. 2022 Apr 20;12:858398. doi: 10.3389/fcimb.2022.858398 (PMC9084186; doi:10.3389/fcimb.2022.858398)
Supplement: Supplementary file 2 [file DataSheet_2.docx]

**Supplement 2.** Procedures for separation, purification, preservation, thawing and staining of peripheral blood mononuclear cells (PBMC)

**PBMC separation and preservation**

Peripheral blood mononuclear cells (PBMC) from each donor were separated using the gradient density separation method. Briefly, 15 mL of whole blood were mixed with equal volumes of phosphate-buffered saline (PBS) solution. The blood-PBS solutions were transferred to 50 mL LeucoSep™ tubes (Greiner Bio-One) previously filled with 15 mL of Lymphoprep™ density gradient medium (STEMCELL Technologies). The tubes were spun for 15 min at 800 x g with the centrifuge’s brakes off. The supernatants were transferred to 50 mL conic tubes, the volumes were completed to 50 mL with PBS and the tubes were spun for 15 min at 250 x g. The supernatants were removed, the cell pellets were resuspended with 10 mL of PBS and the tubes were spun for 10 min at 250 x g. The supernatants were removed, the cell pellets were resuspended in 4 mL of heat-inactivated foetal bovine serum (FBS) and left for 10 min at 4°C. Each tube was then added with 4 mL of medium containing 80% of RPMI 1640 medium and 20% of dimethyl sulfoxide (DMSO). The final composition of the freezing medium was 50% FBS, 40% RPMI 1640 and 10% DMSO. Cells were transferred to 2 mL cryopreservation vials, placed in a Mr. Frosty™ freezing container (Nalgene), stored at a -80°C freezer overnight and finally transferred to liquid nitrogen (-196°C) until performing the assays.

## PBMC thawing

Cryopreservation vials containing the cells were quickly transferred from the nitrogen tank to a 37°C water bath until complete thawing. The cells were immediately poured in 10 mL of a previously warmed up (37ºC) washing medium containing 80% of RPMI 1640 medium and 20% of FBS. The cells were washed twice with the washing medium and, in both washing steps, the tubes containing the washed cells were spun for 10 min at 500 x g. After the second washing step, the supernatant was removed, and the cells were left in 10 mL of RPMI 1640 for 10 min at 37°C with 5% CO_2_. The cells were pelleted again after new centrifugation at 500 x g for 10 min, resuspended in 1 mL of RPMI and assessed for counting and viability using the Nucleo Counter NC-100 device (ChemoMeTec Diagnostics). After that, the PBMC concentrations were adjusted to 2x10^6^ cells/mL and we proceeded to the culture protocol.

## Preparation of the MABSC lysate

Briefly, a *M. abscessus sensu stricto* serovar (CF340) was obtained from a CF patient with diagnosed MABSC infection. This strain was grown in Souton’s medium for four weeks. The bacterial culture was centrifuged (10,000 rpm for 10 min at 4ºC) and remaining bacteria were removed by filtration using a 0.45 µm membrane filter. The culture filtrate underwent X-press disruption five times at maximal force (200 MPa at -20ºC) and then sonicated five times for 5 min each at 80-100 W using a Sonoplus Ultrasonic Homogenizer (BANDELIN electronic GmbH & Co. KG). The lysed cells underwent centrifugation (20,000 rpm for 60 min at 4ºC), after which the supernatant was aspirated and mixed with distilled water (1:1 v/v). The supernatant was again sonicated five times for 5 min each at 80-100 W, and the remaining lysed cells underwent centrifugation (20,000 rpm for 60 min at 4ºC). The resulting supernatant was used as the antigen for the tests. The protein concentration was set at 2.67 mg/mL after analysis in a refractometer (ATAGO Co., LTD). The lysate was aliquoted and stored at -80ºC before preparing the stock solutions (100 µg/mL) for the analyses.

## PBMC culture and stimulation

PBMC (5x10^5^ cells) were cultured in medium containing 80% of RPMI 1640 and 20% of FBS in the presence of the mitogenic stimulant Phytohemagglutinin (PHA, 7.5 µg/mL, Sigma-Aldrich, used as a positive control) and an *in-house* MABSC lysate (10 µg/mL) [13]. Each culture set had a final volume of 1 mL in 12x75 mm polystyrene cytometry tubes, and the cells were cultured for four days at 37°C with 5% CO_2_. The concentrations of PHA and MABSC lysate in the culture set were determined after optimization experiments (data not shown). Unstimulated cells were used as negative controls.

**Cell staining and flow cytometry**

After cultured, the cells were treated with 100 µL of EDTA at 20 mmol/L for 15 min, and vortexed each 5 min to undo cell clots. After adding 3 mL of PBS, the tubes containing the cells were spun for 4 min at 2200 rpm (the same settings were used for the next steps). The supernatants were removed, the pellets were resuspended and unspecific Fc receptors in the cells were blocked with purified human IgG at 50 µg/mL for 5 min. After that, the pellets were stained with fluorophore-conjugated monoclonal antibodies (eBioscience), targeting the surface markers CD45 (PE), CD3 (FITC), CD4 (PE-Cy.7), CD8 (APC), CD19 (eFluor-450), CD45RO (APC-eFluor-780) and CD27 (SuperBright-702), for 20 min, at 4-5°C in the dark. The tubes were spun, the supernatants were removed, and the cell pellets were washed again with 3 mL of PBS. After a last centrifugation step, the supernatants were removed, the pellets were resuspended in 300 µL of PBS and the cells underwent flow cytometric analysis in a 12-color Attune NxT flow cytometer (Invitrogen). For each sample/culture condition, we counted 100,000 events, which were analysed using the FlowJo Software version 10.7.2 (FlowJo, LLC). Briefly, we selected all leukocytes (cells expressing the surface CD45 marker), among which we selected single cells expressing the CD3 (T cell) and CD19 (B cell) surface markers. Within each cell type, we determined the intensity of the anti-MABSC cellular response by calculating the rate of lymphoblast formation upon lymphocyte stimulation with MABSC, given by the percentage of lymphoblasts among the total cells (resting lymphocytes plus lymphoblasts) (**Suppl Figure 1**). The net result was calculated after subtracting the background lymphoblast formation from unstimulated cells. Within the CD3+ lymphoblasts, we determined the rate of lymphoblasts expressing the CD4 and CD8 surface markers, and the rate of cells expressing the memory T cell marker CD45RO within each of these subtypes. Within the CD19+ lymphoblasts, we determined the rate of cells expressing the CD27+ marker, present in plasmablasts and memory B cells. The analysis strategy is better detailed in **Suppl Figure 2**.


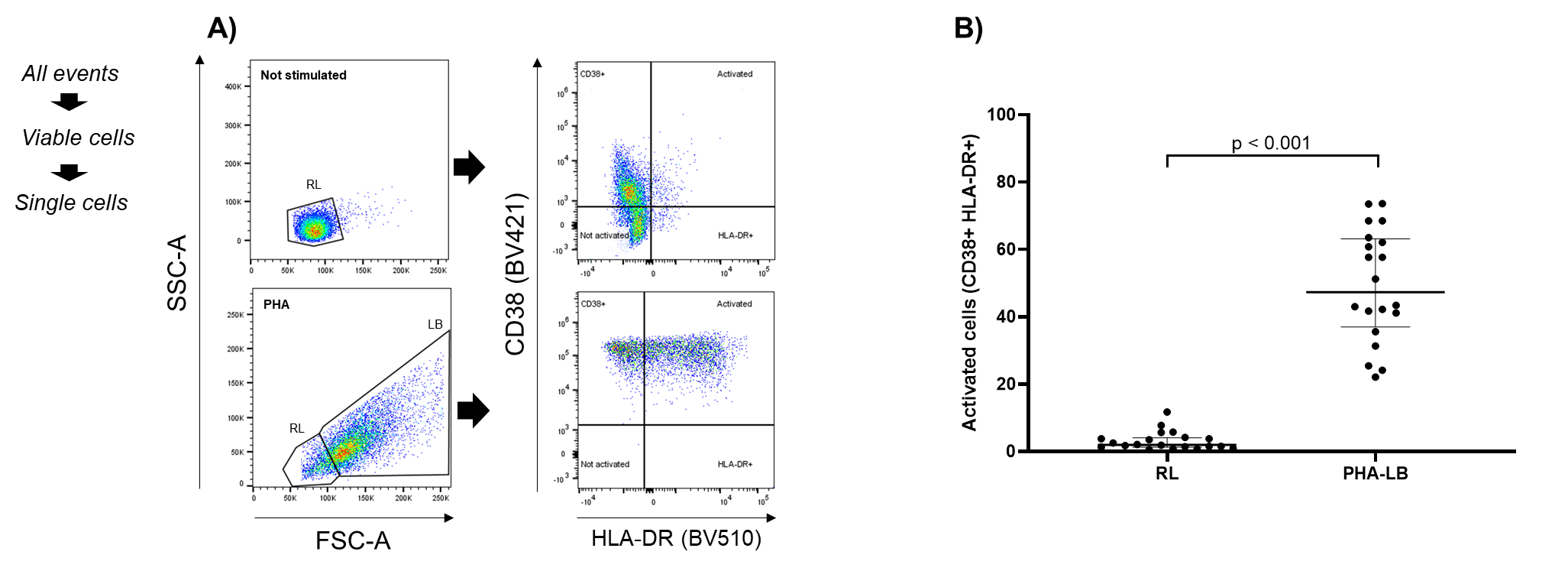


**Supplementary Figure 1**. **(A)** Flow cytometry dot-plots showing activated cells within resting lymphocytes (RL), after culturing PBMC from 20 volunteers under no stimulation, and within lymphoblasts (LB) formed after culturing the same PBMC under stimulation with phytohemagglutinin (PHA). After RL and LB gating, activated cells were gated as cells expressing both CD38 and HLA-DR. **(B)** Rate of activated (CD38+ HLA-DR+) cells within resting lymphocytes (RL, not stimulated) and lymphoblasts formed upon lymphocyte stimulation with PHA (PHA-LB). The middle bold lines indicate the median, and the lower and upper lines indicate the first and third quartiles, respectively. Statistical significance was assessed using the Mann-Whitney test.


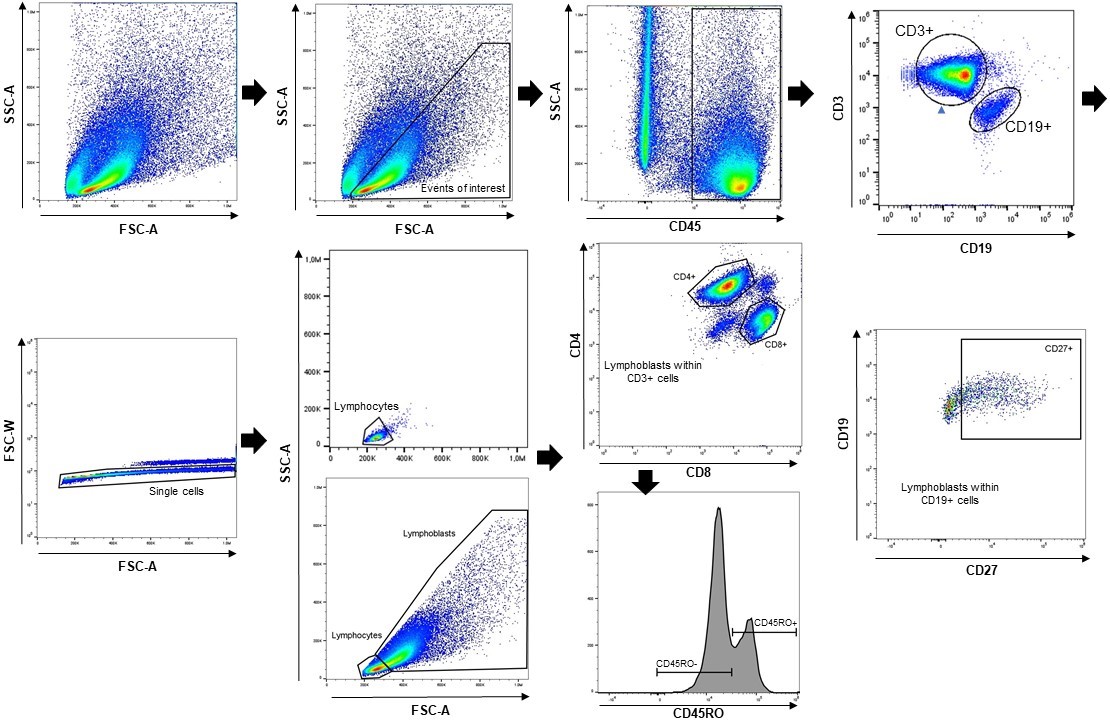


**Supplementary Figure 2**. Flow cytometry gating strategy used to determine the cell populations. The arrows indicate the gating sequence. The first graph shows all the cells sorted according to their size (forward scatter, FSC-A) and granularity (side scatter, SSC-A). Within all events, the events of interest were selected. Within the events of interest, we selected the leukocytes (cells expressing the CD45 surface marker). Within the leukocytes, we selected all the CD3+ (T) and CD19+ (B) cells. Within each cell subtype, we selected the single cells, in order to remove doublets. Using a plot of an unstimulated sample, we defined the gate for the resting lymphocytes, and, using a plot of a sample stimulated with the PHA mitogen, we defined the gate for lymphoblasts. The rate of lymphoblast formation (Lymphoblasts / Lymphocytes + Lymphoblasts) is a marker of the lymphocyte proliferation intensity. After defining these gates, we selected the lymphoblasts within CD3+ and CD19+ cells. After subtracting the background lymphoblast formation in the samples, we selected the CD4+ and CD8+ cells within the CD3+ lymphoblasts, and cells expressing the memory B cell marker CD27 within the CD19+ lymphoblasts. Within CD4+ and CD8+ lymphoblasts, histograms were used to determine the rate of cells expressing the memory T cell marker CD45RO.

**C/BCG-**

**C/BCG+**

**CF/MAC**

**CF/NTM-**

**CF/MABSC**

**Concentration in plasma (pg/mL)**

**IFN-γ**

**TNF-α**

**IL-2**

**IL-17**

**CD40L**

**IL-4**

**IL-5**

**Supplementary Figure 3**. Dot-plots showing the variation in the plasma concentrations (pg/mL) of IFN-γ, TNF-α, IL-2, IL-17, CD40L, IL-4 and IL-5 in CF patients with history of MABSC infection (CF/MABSC), CF patients with history of MAC infection (CF/MAC), CF patients without history of NTM infection (CF/NTM-), non-CF controls vaccinated with BCG (C/BCG+) and non-vaccinated controls (C/BCG-). The extremities of the lines above the plots indicate significant differences between non-stimulated (NS) samples whole blood samples and samples stimulated with MABSC lysate (MABSC) within the group, with *p ≤ 0.05 and **p ≤ 0.01.
